# High-Order Visual Processing, Visual Symptoms, and Visual Hallucinations: A Possible Symptomatic Progression of Parkinson's Disease

**DOI:** 10.3389/fneur.2018.00999

**Published:** 2018-11-27

**Authors:** Kelsey Barrell, Britta Bureau, Pierpaolo Turcano, Gregory D. Phillips, Jeffrey S. Anderson, Atul Malik, David Shprecher, Meghan Zorn, Edward Zamrini, Rodolfo Savica

**Affiliations:** ^1^Department of Neurology, University of Utah, Salt Lake City, UT, United States; ^2^Department of Neurology, Mayo Clinic, Rochester, MN, United States; ^3^Department of Radiology, University of Utah, Salt Lake City, UT, United States

**Keywords:** Parkinson's disease, visual symptoms, visual hallucinations, visual misperceptions, gray-matter volume, REM sleep behavior disorder, cognitive impairment

## Abstract

**Objective:** To determine whether Parkinson disease (PD) patients with (VH) have different clinical characteristics and gray-matter volume than those with visual misperceptions (VM) or other visual symptoms (OvS).

**Background:** The spectrum of visual complaints in PD is broad and complex.

**Methods:** We conducted a retrospective chart review of 525 PD patients to identify the frequency of visual symptoms and the association with clinical and radiological features. Brain volumetric MRI data was analyzed using multivariate logistic regression to differentiate cases with and without visual symptoms.

**Results:** Among 525 PD cases, visual complaints were documented in 177 (33.7%). Among these, 83 (46.9%) had VH, 31 (17.5%) had VM, and 63 (35.6%) had OvS (diplopia, blurry vision, photophobia, dry eyes, and eye pain or soreness). When compared to OvS, patients with VH had significantly higher age, duration of disease, rate of REM sleep behavior disorder, and cognitive impairment. Visual hallucinations patients had decreased age-adjusted volumetric averages in 28/30 gray-matter regions when compared to PD without visual symptoms and 30/30 gray-matter regions when compared to VM patients.

**Conclusions:** Visual symptoms in PD may represent a spectrum from OvS to VM to VH, with progression of the latter associated with older age, duration of disease, presence of REM sleep behavior disorder, cognitive impairment, and decreased gray-matter volume.

## Introduction

Parkinson disease (PD) is a degenerative disorder that recognizes the abnormal aggregation and deposition of alpha-synuclein in the nervous system as the pathological hallmark. Classically, the primary target of degeneration was presumed to be the basal ganglia, causing predominantly motor disturbances. However, it is now evident that non-motor symptoms (NMS) such as autonomic dysfunction, sleep disorders, pain and sensory disorders, mood disturbances, and cognitive complaints are critical manifestations of the neurodegenerative process causing PD (1, 2). NMS of PD have been shown to have a larger negative impact over the health-related quality of life compared to PD-specific motor symptoms (3). Among NMS, visual complaints are a group of symptoms that are common and still poorly understood. Patients with PD may report or further examinations may reveal the presence of blurry vision, double vision, dry eyes, difficulty with contrast recognition, diplopia, visual misperceptions (VM), and visual hallucinations (VH) among others (4, 5). In particular, visual symptoms such as VM and VH are commonly present in alpha-synucleinopathies such as Dementia with Lewy Bodies (DLB) or Parkinson's disease Dementia (PDD) along with idiopathic PD patients. Interestingly, alpha-synuclein has been also reported to be deposited in the retina (6). Deterioration of visual function could possibly be caused by deficiencies of dopamine in the retina, unusual eye movements, decreased blinking rate or increased frequency of nuclear, and posterior sub-capsular cataract in the PD patients (5). This further highlights the role of visual symptoms as a possible clinical biomarker (7–9).

The clinical definition and diagnosis of VM and VH is still challenging. Although VM are considered benign phenomena (9), it is not clear whether they are harbingers for the future development of VH or represent a separate disease phenotype.

Although a number of studies have explored VM and VH, there are not clear data comparing the clinical, radiographic, and phenotypic characteristics of VH with VM. Indeed, a number of studies have explored the volumetric differences of cortical and subcortical structures in PD (10, 11). Nevertheless, the differences in the cortical and subcortical atrophy in PD patients with VM and VH have not been clearly defined. The aim of our study is to fill in this gap of knowledge by studying the clinical and phenotypic characteristics of PD cases affected by VM and VH and determining whether the presence of VM and VH alone or in conjunction with MRI volumetric measures can predict the progression of disease.

## Methods

### Ascertainment

We performed a retrospective chart review to identify all the patients affected with visual symptoms in PD cases. Patients were identified from a movement disorder clinic at the University of Utah Hospital and Clinics in Salt Lake City, Utah; the sole movement disorder center in a 5-state area. Our study was approved by the University of Utah's Institutional Review Board. We only included patients that were diagnosed with PD by a movement disorder specialist (DS) based on the Queen Square Brain Bank criteria (12). We reviewed the medical records of 572 PD patients seen by three providers (DS, MZ, and RS) from January 1st 2011 to December 31st 2014 searching for the presence of NMS (including hallucinations) in the clinical history. The clinical history template was a consistent collection of clinical questions been developed to ensure the completeness of clinical information. These included all motor and NMS. Exclusion criteria included charts with insufficient information, patients lost to follow-up, or patients with an unclear diagnosis. Forty-seven charts met the exclusion criteria, leaving 525 patients in our final sample. The inclusion criteria involved any PD patient who had experienced a visual symptom in one of their last three clinic visits. A neurologist (KJS) reviewed the 525 medical records to determine which patients met the inclusion criteria. One hundred and seventy-seven patients reported visual complaints, which included blurred vision, dry eyes, double vision, VH, or VM. We collected demographic information and clinical characteristics of each patient with PD and visual complaints [age, sex, age-of-onset, type of visual complaints, RBD, cognitive complaints, and use of dopamine agonists (Das)]. We took advantage of the data collected in the medical records of the movement disorders clinic that specifically addressed cognitive complaints, visual complaints, psychiatric complaints, and RBD (defined using the Mayo Clinic Questionnaire) (13). When available, the Montreal Cognitive Assessment (MoCA) was used to define and characterize the presence of cognitive complaints (MoCa score < 26); if unavailable, we used the clinical diagnosis determined by the clinician.

We differentiated between hallucinations and VM (or illusions) by using a structured set of questions that were adopted in the review of the medical records. Hallucinations were defined as a perception of an object (in this case visual) in the absence of an external stimulus (14). In comparison, VM involved identifiable external stimuli (in this case visual) that were integrated incorrectly resulting in a transient phenomenon including shadows, movement, presence, flashing figures, distortions, or corner of eye phenomena. If visual symptoms were only reported in the setting of acute delirium due to a medical illness, they were excluded from the study. We excluded all the cases that reported cataracts diagnosis, cataract surgery, macular degeneration, or had a sudden decline of visual acuity secondary to trauma, presbyopia, or other ophthalmologic reasons.

We also reviewed the available brain MRIs in order to study the volumetric structural changes among the patients with different visual symptoms. Eighty two of our 177 patients (46.3%) had a brain MRI in our hospital and clinics or at an outside institution and were scanned into PACS (picture archiving and communication system). The MRI was performed according to the clinician judgment for clinical or diagnostic reasons. In a minority of cases, MRI scan included a magnetization-prepared rapid acquisition gradient echo (MPRAGE) sequence with spatial resolution of 2 mm isotropic voxels or better. Among patients in our sample, we obtained MRI scans of sufficient quality for volumetric analysis from 11 patients with VH, 4 patients with VM, and 20 patients without VH or misperceptions.

Cortical reconstruction and volumetric segmentation was performed with the FreeSurfer Image Analysis Suite, which is documented and freely available for download online (http://surfer.nmr.mgh.harvard.edu/). The technical details of these procedures are described in prior publications (15, 16). Briefly, this processing includes motion correction and averaging of multiple volumetric T1-weighted images (when more than one is available), removal of non-brain tissue using a hybrid watershed/surface deformation procedure, automated Talairach transformation, segmentation of the subcortical white matter and deep gray matter volumetric structures (including hippocampus, amygdala, caudate, putamen, ventricles) (15, 16), intensity normalization (17), tessellation of the gray matter-white matter boundary, automated topology correction (18), and surface deformation following intensity gradients to optimally place the gray/white and gray/cerebrospinal fluid borders at the location where the greatest shift in intensity defines the transition to the other tissue class (19). This was followed by parcellation of the cerebral cortex into units with respect to gyral and sulcal structure using the Destrieux atlas (20).

### Statistical analysis

Consistent with our study design, we calculated median, 25th percentile, and 75th percentile—as well as mean and standard deviation—for continuous variables, and frequency and percent for categorical variables. To compare groups, we report *p*-values from the Mann–Whitney *U*-test (continuous variables) and Fisher's exact test (categorical variables). Multivariate logistic regression was performed to explore the correlation of age, age at disease onset, duration of disease, RBD, cognitive impairment, and DAs between clinical groups while adjusting for covariate gender. To avoid collinearity, we did not include covariates age, age at disease onset, or duration of disease simultaneously in a logistic regression model. All statistical analyses were performed at the conventional two-tailed alpha level of 0.05 using Stata-12.1 statistical software (StataCorp LP). Brain gray matter volumetric measurements were compared between groups using a two-tailed *t*-test in Matlab software package (Mathworks).

### Ethics approval

The Institutional Review Board of the University of Utah approved this study. Patients (or their representatives) gave written consent for the use of their medical information.

## Results

### Demographics

From the 525 charts reviewed 177 (33.7%) met inclusion criteria for having visual symptoms. Table 1 shows the prevalence of visual complaints in our PD sample. 83 (15.8%) patients had VH, 31 (5.9%) patients had VM and 63 (12%) patients had other visual symptoms (OvS) that included diplopia, blurry vision, photophobia, dry eyes, and eye pain or soreness.

**Table 1:** Demographic and clinical features of PD sample.

**Symptoms**	**VH**	**VM**	**OvS**	**Any-visual symptoms**
	**(*n* = 83)**	**(*n* = 31)**	**(*n* = 63)**	**(*n* = 177)**
**AGE**				
-Mean (SD)	72.0 (8.2)	69.2 (10.5)	66.7 (11.4)	69.6 (10.1)
**-**Median (25%, 75%)	73 (68, 78)	71 (62, 77)	65 (58, 74)	71 (63, 76)
-Range (Min, Max)	(43, 90)	(43, 88)	(44, 90)	(43, 90)
**PD AGE ONSET**				
-Mean (SD)	61.8 (10.0)	60.3 (11.3)	59.3 (11.5)	60.6 (10.80)
-Median (25%, 75%)	64 (55, 68)	62 (55, 70)	60 (52, 66)	60 (53, 68)
-Range (Min, Max)	44 (39, 83)	48 (32, 80)	44 (38, 82)	51 (32, 83)
**PD DURATION**				
-Mean (SD)	10.1 (6.0)	8.9 (5.2)	7.4 (5.1)	8.9 (5.6)
-Median (25%, 75%)	9 (5,14)	7 (4, 11)	7 (4, 10)	7 (4, 12)
-Range (Min, Max)	27 (2, 29)	22 (3, 25)	26 (0, 26)	29 (0, 29)
Male Gender, n (%)	60 (72.3)	19 (61.3)	44 (69.8)	123 (69.5)
RBD, n (%)	50 (60.2)	18 (58.1)	24 (38.1)	92 (52.0)
Cogn, n (%)	72 (86.8)	27 (87.1)	42 (66.7)	141 (79.7)
DA ag, n (%)	45 (54.2)	19 (61.3)	29 (46.0)	93 (52.5)

The demographic characteristics of our PD sample are summarized in Table 2; 123 (69.5%) were male. Median age of PD onset was 60 (IR: 53–68) years old without any significant differences across the three groups. The median age of patients with VH was 73 [*p* < 0.0039; Interquartile Range (IR): 68–78], VM was 71 (IR: 62–77) and with OvS was 65 (*p* < 0.003; IR: 58–74). Median PD duration was 9 (IR: 5–14) years in the VH, 7 (IR: 4–11) in VM, and 7 (IR: 4–10) in the OvS group. Those with VH compared to VM had an older median age at onset (64 vs. 63 years), longer median duration of disease (9 vs. 7 years). No significant difference was observed between the three groups regarding Dopamine agonists.

**Table 2:** Percentage of visual complaints in the study population, *n* = 525.

	**N**	**Percentage in visual symptoms group (177)**	**Percentage in entire study population group (525)**
VH	83	46.9%	15.8%
VM	31	17.5%	5.9%
^*^OvS	63	35.6%	12.0%

* *diplopia, blurry vision, photophobia, dry eyes, eye pain*.

### RBD and cognitive decline

OvS patients had a lower frequency of RBD (38.1%) as compared with the two other groups (*p* = 0.008). Those with VH and VM had a higher frequency of RBD as compared with OvS (60.2 vs. 58.1%) (*p* = 0.050).

Cognitive decline was different across the three groups (*p* = 0.003); in particular, OvS patients had a lower frequency of cognitive decline compared with the two other groups (*p* = 0.008). On the other hand, the patients with VH had a higher frequency of cognitive decline compared with the other two groups (*p* = 0.039).

In addition, we observed that the presence of RBD is associated with higher odds of developing VH rather than OvS (*p* = 0.012). In addition, cognitive complaints were more present in patients with VM and VH when compared with OvS, respectively (*p* = 0.047 and *p* = 0.005), but there was not a significant difference between VM and VH (*p* = 1.00).

### Comparison between each group of patients with visual symptoms (VH vs. VM vs. OvS)

We further compared the groups individually to each other. Patients with VH had a significantly older age than patient with OvS (*p* = 0.001), but there was not a significant difference between VH and VM (*p* = 0.2926).

The age of onset of PD was not different across the different groups. On the contrary, the patients with VH had a longer duration of PD compared with OvS (*p* = 0.0073) but not with VM patients (0.3951).

### Volumetric comparison of brain MRI between groups

Eighty two of our sample patients (14.9%) had clinically indicated brain MRI. Those with illusions were more likely than those with hallucinations to have had MRI (60.9 vs. 25.9%). For volumetric analysis, we included only MRI scans performed using a standard MPRAGE sequence at our institution for internal consistency of methodology. Only 15 brain MRI studies from our sample were obtained: 8 from patients with VH, 4 from patients with VM and 3 from patients with both VH and VM. These were compared to a control group of 20 MRIs from patients with PD who had no visual symptoms.

**Figure 1:**
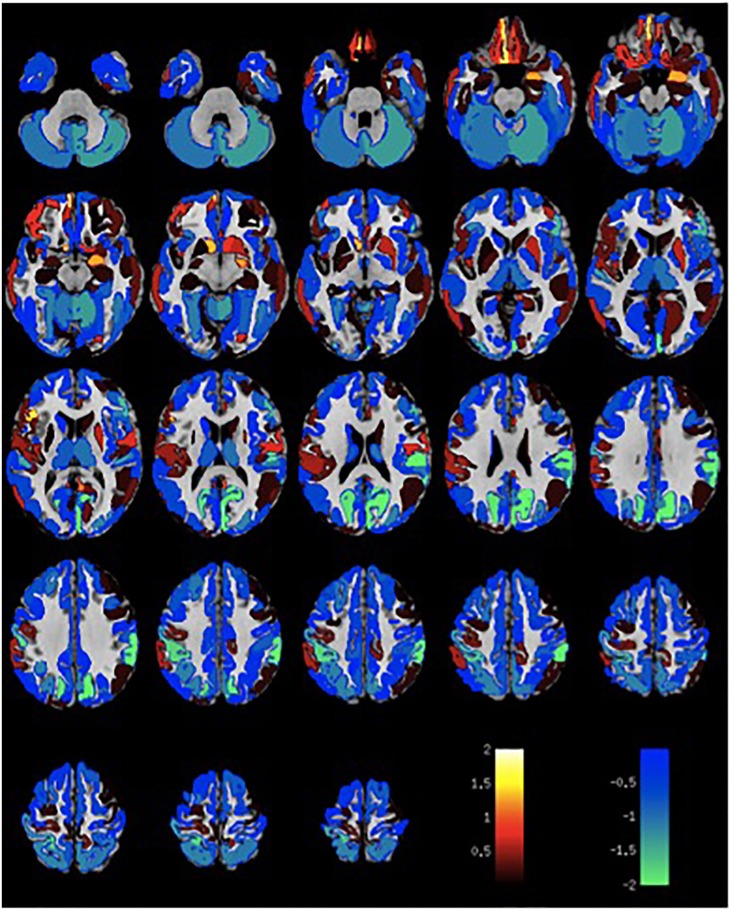
Differences in gray matter volume in patients with visual hallucinations and Parkinson Disease compared to patients with Parkinson Disease without hallucinations or misperceptions. Color scale shows *t*-statistic for differences in gray matter volume in cortical regions of the Destrieux atlas and 14 subcortical regions with automated FreeSurfer segmentation.

**Figure 2:**
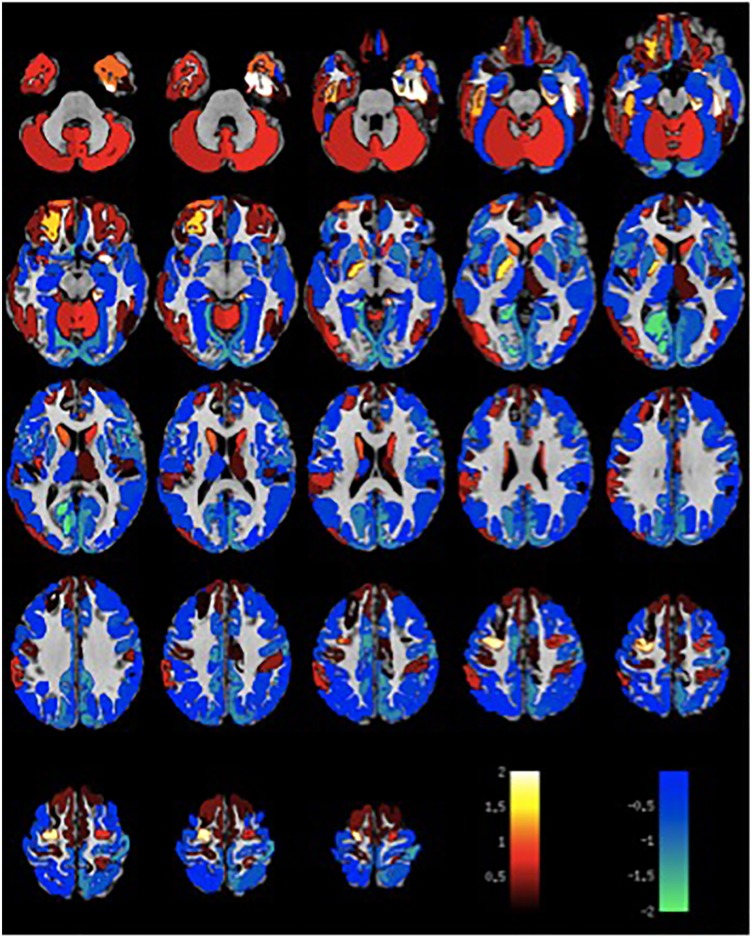
Differences in gray matter volume in patients with visual misperceptions and Parkinson Disease compared to patients with Parkinson Disease without hallucinations or misperceptions. Color scale shows *t*-statistic for differences in gray matter volume in cortical regions of the Destrieux atlas and 14 subcortical regions with automated FreeSurfer segmentation.

Given the small sample size, volumetric differences were not significant with false discovery rate multiple comparison corrections across brain regions. Nevertheless, the spatial distribution of volumetric changes was informative, particularly for the patient cohort with VH. Cortical volumes were decreased relative to control patients with Parkinson's disease but without VH or misperceptions for most cortical regions (Figure 1). Cortical volume loss was greatest along the intraparietal sulcus and precuneus bilaterally, as well as in the bilateral cerebellar hemisphere. Smaller differences in cortical volumes were noted in patients with VM (Figure 2). No significant differences were noted in the presence of visual symptoms using different antiparkinsonian agents; however, the vast majority of our patients used levodopa at maximum effective doses rather than other dopaminergic drugs.

## Discussion

Our study assessed the clinical and radiographic characteristics of visual symptoms (VH, VM, and OvS) in a clinical series of PD patients from a tertiary movement disorder center. Our findings highlight the diversity of visual symptoms in PD and underscore the complex interaction between visual symptoms and several demographic and clinical NMS of PD. In particular, the patients with PD affected by hallucinations and misperceptions were more likely to have RBD. This supports the strong association of RBD with alpha-synuclein deposition and predates the onset of PD for several decades; therefore it would be expected to be found to a similar extent in all alpha-synucleinopathies (21, 22). In addition, our findings may indicate that patients presenting both RBD with VM and VH represent a more aggressive phenotype of alpha-synucleinopathy. Clinical differences were less evident between VH and VM; both groups had a significantly longer duration of PD as well as increased presence of RBD and cognitive complaints compared to those with OvS. The small number of patients in the VM group somewhat limited the ability to draw statistical or clinically significant differences. No significant difference in medications was observed in the three groups as well there was no difference regarding the on/off status of the cases affected by PD.

A number of studies have explored the role of visual symptoms in PD (23–25). The degeneration of dopaminergic neurons occurring in the *substantia nigra* seems also to occur in dopaminergic neurons of the retina, where it functions as a modulator guiding visual signal transmission (26). Visual symptoms stem from two different processes. There are basic visual processes including visual acuity, spatial contrast sensitivity, and color discrimination, which are more likely affected by retinal processes, as well as higher-order visuoperceptual systems: visuospatial processing, visuospatial problem solving, and spatial working memory, which are involved with the degeneration of central dopaminergic pathways (nigrostriatal, mesolimbic, and mesocortical) (27).

The mechanism of VH in PD is still unclear, but traditionally VH have been thought to result as a side effect of dopaminergic or even cholinergic or serotonergic medications (28). However, it is also hypothesized that VH closely correlate with impaired visual acuity. It is hypothesized that poor visual acuity is a risk factor for VH, and if this is the mechanism, proper corrections can be made to stop Parkinson's patients' VH (28).

From a pathophysiology standpoint, many different theoretical models have been postulated to explain the development of VH in PD (29); on the other hand, many of the different theories are sharing attentional and perceptual impairment as common affected functions of VH in PD (30). The role of attentional-control networks has been explored with functional MRI studies, showing the importance of the interplay between the different subdivisions of the network in patients that develop VH in PD (31).

While the later involvement of central dopaminergic pathways correlates with progression to Braak stage 3 (or 4), the involvement of the retinal dopaminergic pathway is not explained by Braak hypothesis (32). This divergent neuro-anatomic involvement is manifest in the wide variety of visual complaints reported in PD. For example, VH are most consistent with involvement of central dopaminergic pathways, due to Lewy-body pathology and nerve cell loss in the ventral-temporal regions of the brain (33). Interestingly, evidence suggests that structural changes in the fovea of patients with PD with retinal optical imaging (OCT) showed morphological changes of volume loss in the retina such as retinal thinning (8, 34, 35). To our knowledge, VM have never been systematically compared to VH in Parkinson's disease, although VM have been studied using different nosology nomenclature (minor hallucinations) that are considered a form of hallucination rather than a misidentification of a stimulus (36, 37).

The frequency of visual symptoms that we report in our study is much lower than previously described. The presence of VH in PD has been reported with an extremely wide range of occurrence from 6 to 87% (38); however, some recent studies observed that up to 50% of patients had VH (33, 38). This variability in the prevalence of VH reflects the uncertainty in the clinical definition and the diagnostic accuracy of VH. As evidence, Williams et al. found that the prevalence of hallucinations nearly doubled with the use of a structured review (39). Therefore, the low prevalence of VH in our study (15.8%) may be due to the lack of a structured interview.

In our study, we observed a number of differences between VH, VM, and OvS. Based on the different clinical characteristics found in the VH and OvS groups, we support OvS as a distinct entity from VH. Though there were some significant differences between VM and OvS, VM and VH had fewer recognizable differences. Unfortunately the small sample size of patients with VM prevents us from drawing firm conclusions; however, VM seem to represent an intermediate stage between the two other samples. A number of studies suggest that the presence of VM may be a more specific symptom of PD as compared to the presence of VH. It has been reported that visual illusions (synonymous with VM) were statistically more likely to occur in PD vs. control (17 vs. 0%), respectively, whereas simple VH were not (10.2 vs. 8.9%) (38). The benefit of studying VM as opposed to VH is the lower likelihood of VM being a side effect secondary to medication, acute medical illness, or a primary psychiatric diagnosis.

The identification of alpha-synuclein deposition as a hallmark of PD, PDD, and DLB has obscured the differentiation between these three diseases, raising the question of whether they represent different points along the same continuum.

Structural imaging found that patients with parkinsonism (PD, PDD, and DLB) who experienced delusional misidentification and those with VH had volume loss within attentional regions of the parietal lobe (40) including intraparietal sulcus and precuneus (41). This may suggest that visual symptoms involve disrupted integration of dorsal visual attentional processing or control of attention to visual percepts. A recent study focusing on the neural correlates of minor hallucinations (passage and presence hallucinations) utilizing voxel-based morphometry found that patients with minor hallucinations had more gray-matter volume loss in multiple regions, most dramatically in the precuneus, compared with controls (11). These findings are in line with our results that support a role for the precuneus and the default network in the development of VH. Interestingly, in light of the evidence of structural changes in the fovea and retina of patients with PD, we may speculate that the brain volumetric changes that we observed correspond or correlate with retinal or foveal changes. Typically, VM and VH are characteristic of PD and DLB but can also be seen in other types of parkinsonism, such as Multiple System Atrophy and progressive supranuclear palsy (42). The presence of VH and their relationship with volumetric brain changes seen at the MRI in specific areas of the brain, can help make a differential clinical diagnosis amongst the Lewy body disorders; for example, a possible to fiber tracts connecting the nucleus basalis of Meynert to the cerebral cortex may contribute to VH in PD, possibly due to a loss of cholinergic innervation. Thus, additional mechanisms and hypotheses can be evoked to explain the generation of high-order visual symptoms in PD (43). Further studies are needed to correlate the eye-findings of OCT with the brain structural changes across the different groups of patients with alpha synucleinopathies.

We propose a possible spectrum of progression of disease and severity of visual symptoms that can be observed in patients affected by synucleinopathies. There can be a possible continuum of progression and severity from a specific visual symptoms to formed hallucinations: the visual symptoms may indeed correlated with a more severe and more diffuse deposit of Lewy Bodies in the patients affected by synucleinopathies.

## Limitations

Our study has a number of limitations. First, the retrospective nature of the study is prone to recall bias. Thus, it is possible that some information was not reported or elicited by the clinician. The presence of a recall bias and under- reporting of visual information is, unfortunately, an inherent part of the study design and warrants caution in the interpretation of the data. However, over the past 3 years, the clinical notes have become standardized, including key NMS (visual complaints, RBD, cognitive complaints, and DA agonists), improving the data collection. Second, the small number of MRI studies limits the volumetric analysis. Additionally, MRIs were obtained at multiple locations with slightly variable techniques, which frequently were not compatible with post-procurement processing. Prior to 2010, very few MRIs included high spatial resolution sequences needed for volumetric processing. To achieve a homogenously acquired imaging sample, only a relatively small minority of cases could be included; thus, the findings of the volumetric analyses should be considered preliminary, hypothesis-generating, and warranting caution in the interpretation. Further studies are definitively needed, involving multimodal imaging, to differentiate between such different visual symptoms in PD.

## Conclusion

Although there is a large spectrum of visual complaints in PD patients, there were some clear clinical distinctions between PD patients with VH and OvS supported by different prevalence of RBD and cognitive complaints as well as a different age of onset and duration. Differences between VH and VM were not statistically significant; rather, VM seemed to be intermediate to VH and OvS in regard to all demographic and clinical features except DA agonists and cognitive complaints. This observation may reflect distinct clinical phenotypes at different stages along the clinical spectrum of alpha synucleinopathies. Future studies are needed to support the difference between VM and VH and the possible role of VM as a predictor of phenotypic characteristics and prognosis of PD.

## Ethics statement

This study was carried out in accordance with the recommendations of the Institutional Review Board of the University of Utah Medical Center with written informed consent from all subjects or their authorized representatives. All subjects gave written informed consent in accordance with the Declaration of Helsinki. The protocol was approved by the Institutional Review Board of the University of Utah.

## Author contributions

KB data collection, editing, first draft. BB and PT editing and data management. GP data analyses. JA and AM data analyses and cases collection. DS, MZ, and EZ data collection, editing. RS study conception, data analyses, data collection, editing.

### Conflict of interest statement

The authors declare that the research was conducted in the absence of any commercial or financial relationships that could be construed as a potential conflict of interest.

## Abbreviations

DA: Dopamine agonists
DLB: Dementia with Lewy bodies
IR: Interquartile range
MoCA: Montreal cognitive assessment
MPRAGE: Magnetization prepared rapidly acquired gradient echo
NMS: Non-motor systems
OCT: Retinal optical imaging
OvS: Other visual symptoms
PD: Parkinson disease
PDD: Parkinson disease dementia
RBD: REM sleep behavior disorder
VH: Visual hallucinations
VM: Visual misperceptions.
